# A Comparative Study of Top-Down and Bottom-Up Carbon Nanodots and Their Interaction with Mercury Ions

**DOI:** 10.3390/nano11051265

**Published:** 2021-05-12

**Authors:** Federico Bruno, Alice Sciortino, Gianpiero Buscarino, Maria Laura Soriano, Ángel Ríos, Marco Cannas, Franco Gelardi, Fabrizio Messina, Simonpietro Agnello

**Affiliations:** 1 Dipartimento di Fisica e Chimica—Emilio Segrè, Università degli Studi di Palermo, Via Archirafi 36, 90123 Palermo, Italy; federico.bruno@unipa.it (F.B.); alice.sciortino02@unipa.it (A.S.); gianpiero.buscarino@unipa.it (G.B.); marco.cannas@unipa.it (M.C.); franco.gelardi@unipa.it (F.G.); 2 Advanced Technologies Network Center, Università degli Studi di Palermo, Viale delle Scienze Ed. 18/A, 90128 Palermo, Italy; 3 Department of Analytical Chemistry and Food Technology, Faculty of Chemical Science and Technology, University of Castilla-La Mancha, 13071 Ciudad Real, Spain; angel.rios@uclm.es; 4 Regional Institute for Applied Chemistry Research (IRICA), 13071 Ciudad Real, Spain

**Keywords:** carbon dots, optical nanomaterials, sensing

## Abstract

We report a study of carbon dots produced via bottom-up and top-down routes, carried out through a multi-technique approach based on steady-state fluorescence and absorption, time-resolved fluorescence spectroscopy, Raman spectroscopy, infrared spectroscopy, and atomic force microscopy. Our study focuses on a side-to-side comparison of the fundamental structural and optical properties of the two families of fluorescent nanoparticles, and on their interaction pathways with mercury ions, which we use as a probe of surface emissive chromophores. Comparison between the two families of carbon dots, and between carbon dots subjected to different functionalization procedures, readily identifies a few key structural and optical properties apparently common to all types of carbon dots, but also highlights some critical differences in the optical response and in the microscopic mechanism responsible of the fluorescence. The results also provide suggestions on the most likely interaction sites of mercury ions at the surface of carbon dots and reveal details on mercury-induced fluorescence quenching that can be practically exploited to optimize sensing applications of carbon dots.

## 1. Introduction

Carbon nanodots (CDs) are a nanometric form of carbon characterized by excellent optical properties and a great versatility for applications in nanotechnology, such as in optoelectronics, photocatalysis, bioimaging, and sensing [1,2,3,4]. CDs are nanoparticles with a size of 10 nm or less, characterized by a carbon-rich core highly functionalized by nitrogen- and oxygen-containing functional surface groups, and displaying strong optical absorption and tunable emission transitions in the visible range. While the nature of these optical transitions is widely debated in the literature, most authors agree that they most likely depend on the groups present on the surface [5,6,7,8]. However, the mechanism of CD emission remains very debated, complicated by the variability and complexity of the possible CD core and surface structures. Generally speaking, the main possible emission mechanisms can be classified in at least two types: pure surface effects, where both excitation and emission depend on molecular-like chromophores on the surface of the dots [7,8]; and core-surface coupling, where the excitation is initially generated in the core and later transferred on the surface, on which radiative recombination occurs [9,10].

Beside the variety of possible applications, other key benefits of CDs with respect to other nanomaterials are the ease of synthesis, the high dispersibility in polar solvents like water, and the huge variety of precursors, such as small molecules, raw materials, organic waste, and the availability of different synthetic approaches, which provide specific tuning of their properties and can be divided into two large families: bottom-up and top-down approaches [1,2,3,4,6,11,12,13,14]. The bottom-up synthesis of CDs proceeds through the carbonization of small molecular precursors. In particular, one of the most widespread bottom-up synthesis routes produces CDs from a mixture of citric acid and a nitrogen-containing molecule such as urea [11,12,15,16,17,18,19]. When these molecular precursors are pyrolyzed by microwaves or in autoclave, the synthesis readily produces a black nanopowder of CDs, highly dispersible in water and displaying remarkable fluorescent properties. Depending on the conditions, these CDs can display blue [20], green [21], or red emissions [21,22], although an extensive purification is often needed to isolate CDs from molecular intermediates produced during the synthesis [23]. Concerning the top-down approaches, the precursors are extended carbon structures or nanostructures, such as graphite, amorphous carbon, carbon fibers, nanotubes, and fullerene, which are fragmented chemically or physically in order to obtain very small carbon nanoparticles [13,24,25,26]. One example is the oxidation of graphite in a highly acidic environment [27,28], which makes it possible to break up the bulk precursor and, at the same time, functionalize the surface, thus giving the typical optical properties of CDs. Top-down synthetic routes are generally much more complex and time-consuming, and very often yield CDs with lower emission quantum yields. However, it has been argued that they allow for much better structural control and purity of the end-products [29,30].

In principle, one would expect CDs to display a range of common properties independently of the synthesis route, although the fundamental emission mechanisms are not necessarily expected to be the same for bottom-up and top-down CDs, considering that the latter should generally host much better-defined core structures. Surprisingly, there is a lack in the literature of direct side-to-side comparisons between CDs obtained by bottom-up and top-down routes, hindering the possibility of highlighting such similarities or to reveal any fundamental differences.

Sensing is possibly among the most important applications of CDs, and certainly one of the most widespread [31,32,33]. Due to the excellent optical properties, the strong sensitivity of the fluorescence to the external environment [34,35], and the capability of CDs to behave as efficient electron donors [36,37,38], CDs are often proposed as detectors of various harmful substances, for example, heavy metals such as mercury [39,40,41,42], copper, and iron [43,44,45,46]. Therefore, a consistent effort is being made to increase the sensitivity of CDs to one or more of these analytes. However, only a few studies have tried to investigate the interactions of CDs with metal ions at a more fundamental level [38,47,48,49], trying for instance to discriminate static from dynamic emission-quenching mechanisms, or to disclose the interaction pathways of the ions with particular surface groups present on the dots. Besides, these interactions can be used, in reverse, as a way of probing the fluorescent groups of CDs and learning about the emission mechanisms.

In this article we present a comparative study of two different types of CDs produced by a bottom-up (BU) and a top-down (TD) method. After characterizing the structure and the optical features of the prepared CDs, we tested their interaction with mercury ions and found that these latter behave as strong quenchers of CD emission; in fact, this important pollutant was detected by two types of dots and was used as the analytical tool to probe diverse behaviors and sensitivities by optical instrumentation. The results provide useful information to start a library of CDs prepared from BU and TD methods and highlight similarities and differences in sensing based on their interaction paths with ions.

## 2. Materials and Methods

**Synthesis of CDs**. The CDs were prepared using two different synthesis approaches. The BU CDs were prepared by pyrolytic decomposition in a 240-mL Parr reactor (model 4840, Parr Instrument, Moline, IL, USA) of an ethanolic solution of citric acid (Sigma Aldrich, Munich, Germany) and urea (Sigma Aldrich) in a molar concentration ratio of 1:1. The reaction was conducted at a temperature of 200 °C and a reactant vapor pressure of 14 bars for about three hours. The endproduct of the reaction was purified by size exclusion chromatography (SEC) through Sephadex G25 (Sigma Aldrich) [15]. To this aim, Sephadex was first activated in water to create a gel, and then packed in a 30-cm-long column with a diameter of 23 mm. Successively, the raw product of the synthesis was processed through the column using water as an eluent. This purification procedure was chosen to eliminate by-products of the pyrolysis reaction, such as small molecular impurities and large aggregates, and to isolate the fraction yielding the strongest green fluorescence by in situ monitoring the emission of the eluted part using the excitation by a 405-nm laser diode.

For the preparation of TD carbon dots, about 500 mg of carbon nanopowder (Sigma Aldrich—nanoparticles with a nominal average size <100 nm) was allowed to react in a reaction flask with a mixture of H_2_SO_4_ (Sigma Aldrich, 96%) and HNO_3_ (Sigma Aldrich, 65%) in a 3:1 ratio [13]. The reaction, strongly exothermic, was carried out at 140° for two days under heating and stirring. As a last step, the solution was neutralized with 1 M of sodium bicarbonate, and the supernatant (sample TD) was recovered by centrifugation. The TD nanoparticles were also surface-passivated by the addition of 10 mL of acetone just before neutralization, thus obtaining a second family of top-down nanoparticles, named TDA. Alternatively, another functionalization was performed by taking 10 mL of aqueous solution of TD, which was reacted by heating and stirring with ethylenediamine (EDA), and the resulting product was named TDN. Both the TD, TDA, and TDN samples were extensively purified by dialysis against 1 L of water for 48 h. All the samples were then studied in aqueous solution.

**Steady-State Spectroscopy**. All optical measurements were made at room temperature in a 1-cm quartz cuvette. The solutions of CDs were water diluted in order to have an absorption of less than 0.3 OD. Sensing tests were carried out by adding increasing amounts of mercury ions to these solutions and monitoring the changes in the optical properties.

The absorption spectra were recorded in aqueous solution with a Star Line ULS2048CL-EVO single-beam optical fiber spectrophotometer (Avantes, Apeldoorn, The Netherlands) equipped with dual halogen-deuterium light source) with a spectral range of 200–1200 nm. Photoluminescence (PL) spectra were recorded by a FP6500 spectrofluorometer (JASCO Corporation, Tokyo, Japan), using 3-nm bandwidth in excitation and emission. The system was equipped with a 150 W Xenon lamp source and used a photomultiplier as detector, operating in the range 1.8–4.1 eV. Fluorescence quantum yields were estimated by a comparison procedure with fluorescein reference.

**Time-resolved Spectroscopy**. Time-resolved photoluminescence measurements were recorded by an intensified CCD (Princeton Instruments, Trenton, NJ, USA) camera while exciting the sample by a tunable VIBRANT laser system (Opotek, Carlsbad, CA, USA) providing 5 ns pulses, 10 Hz repetition rate, 10–100 µJ/pulse from an optical parametric oscillator pumped by the third harmonic of a Nd:YAG laser.

**Raman Spectroscopy**. Raman spectra were acquired by a LabRam HR Evolution microRaman spectrometer (Horiba, Kyoto, Japan), under a 325 nm excitation wavelength. The laser power was reduced to avoid any sample degradation. Measurements were acquired with integration times of 5–10 s, averaging 10 spectra, and several independent measurements (typically 5) were acquired per sample in different positions to enhance the statistical significance of the sampling procedure. The Raman signal was dispersed on a grating with 600 lines/mm, calibrated by using the Raman line of a silicon substrate. In these conditions, we obtained a spectral resolution of 2 cm^−1^. The samples were prepared by depositing a drop of CDs solution on a silica or on a silicon slide.

**Attenuated Total Reflection (ATR) Spectroscopy**. ATR spectra were acquired on a Platinum ATR spectrometer (Bruker, Billerica, MA, USA) equipped with a single-reflection diamond crystal. Measurements were carried out at room temperature by depositing a drop of concentrated CD solution in water on the sample holder of the instrument, and letting the solvent dry out before acquiring spectra in the 200–4000 cm^−1^ spectral region. Each measurement was acquired with an integration time of 5–10 s. Measurements were repeated a few times and averaged to further increase the signal-to-noise ratio.

**Atomic Force Microscopy (AFM)**. Atomic force microscopy (AFM) images were acquired on a sample obtained by depositing a drop of an aqueous solution of CDs on a mica substrate having sub-nanometer surface roughness. After drying in a vacuum environment for 2 h in tapping mode, AFM measurements were performed at room temperature by using FastScan Bio and FastScan A probes (Bruker) with a tip radius approximately equal to 5 nm, equipped with a closed-loop piezoscanner (maximum xy range ≈ 34 µm and maximum z range ≈ 3.6 µm) and a four-segment photodetector for cantilever deflection monitoring. The scanner was calibrated by using a 1 µm × 1 µm reference grid. The nominal resonant frequency and spring constant of the probe were 1400 KHz and 18 N/m, respectively. AFM images were obtained with a tip velocity of 20 µm/s and a target amplitude of about 15 nm. The pixel resolution was fixed at about 1000 × 1000 points. Each sample was typically characterized by acquiring five images obtained in different points.

**Sensing tests**. Sensing tests were carried out by adding increasing amounts of mercury chloride (0–40 µM) (Sigma Aldrich) to aqueous carbon dots solutions and monitoring the changes in the fluorescence intensity and lifetime under 440 nm excitation.

## 3. Results and Discussion

We aimed to carry out a side-to-side comparison between CDs synthesized through bottom-up (BU) and top-down (TD) methods, especially in relation to their response to mercury ions. The synthesis of our nanoparticles is described in detail in the experimental section. Typically, a dense surface functionalization of BU-CDs automatically builds up during the synthesis, and their optical response is often very good without the need for further treatments. Thus, BU-CDs are often used in the as-synthesized form, only followed by purification [1,2,3,6,15]. In contrast, the surface groups of TD-CDs typically need to be tailored by post-synthesis functionalization procedures to achieve the desired properties. Here, top-down CDs were used either as-synthesized or passivated by reaction with acetone (TDA) or ethylenediamine (TDN). In this way we obtained four different types of CDs (BU, TD, TDA, TDN) and studied them in detail, as described hereafter.

### 3.1. Morphological and Structural Characterization

**Figure 1:**
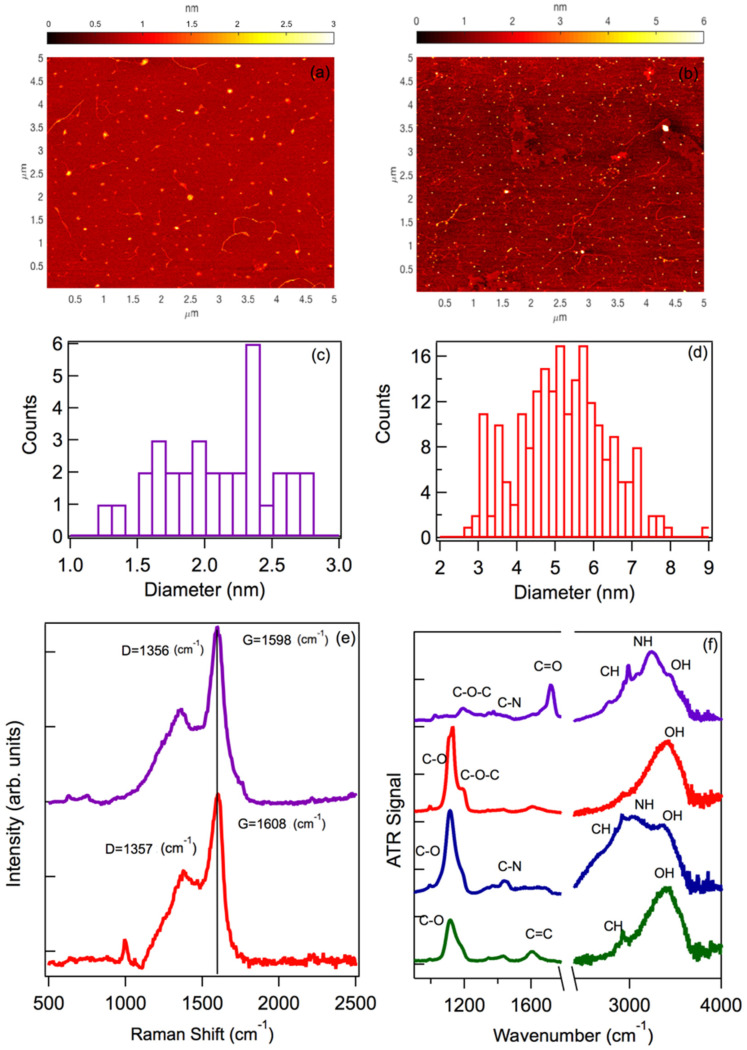
AFM images of representative (**a**) BU and (**b**) TD samples. Particle diameter distribution for the (**c**) BU and (**d**) TD samples. (**e**) Normalized Raman spectra of BU (purple line) and TDA (red line) samples and (**f**) normalized ATR spectra of BU (purple line), TD (green line), TDA (red line), and TDN (blue line) samples. In the latter two panels the spectra were arbitrarily vertically shifted for clarity.

We carried out an extensive structural and morphological characterization of the CDs by AFM, Raman, and ATR measurements. The resulting data highlight both similarities and differences between BU and TD nanodots. Figure 1a,b shows the AFM images obtained from two representative samples, obtained by the two methods, after SEC or dialysis. While both images clearly show the presence of nanoparticles confirming the efficient production of CDs through both synthesis routes, their size distributions, plotted in Figure 1c,d, are quite different. It can be seen that the BU nanoparticles featured an average size of about 2 nm, whereas the top-down nanoparticles showed a much larger average size, about 5 nm. The difference is justified by the different synthetic approaches. Indeed, BU synthesis proceeds through the carbonization of sub-nanometric molecular precursors, from which the reactive and growth processes only allow for the formation of relatively small carbon nanodots. In contrast, top-down synthesis begins from much larger precursors, in this case <100 nm graphite carbon nanoparticles, which are broken up into CDs by the reactive process, but only up to a certain extent. Both size distributions are quite broad, confirming a high structural inhomogeneity typical of most CDs, a property which seems to be independent of the synthesis route.

In Figure 1e, we report the Raman spectra of two representative samples, providing information about the core structure of the nanoparticles. The BU nanoparticles displayed the characteristic G band at 1598 ± 2 cm^−1^ and D band at 1356 ± 2 cm^−1^, well-known as the typical Raman signals of carbonaceous nanomaterials, which thus confirmed the successful carbonization of the reactants into a well-defined nanoparticle [50,51,52,53]. The intensity ratio I_D_/I_G_, calculated to be 0.53, is consistent with a nanomaterial with a highly defective graphitic carbon core [50,51,52,53]. This was confirmed by the displacement of the G and D bands from their usual positions for a carbon nanostructure with perfect sp^2^ hybridization, that were at 1566 and 1364 cm^−1^. Therefore, our nanoparticles displayed a carbon core with mixed sp^2^ and sp^3^ hybridization [51]. The Raman spectrum of the top-down CDs also highlighted the G and D bands, although their positions changed slightly from BU (we found 1608 and 1357 cm^−1^). Again, these shifts imply the presence of a nanocrystalline structure based on graphite, but with an amorphous component, possibly caused by the surface defects and surface passivation, which both contribute to a partial loss of the graphitic component. Notably, the TDA also showed a noticeable Raman band around 1000 cm^−1^, probably related to the surface functionalization of these dots upon acetone addition. Furthermore, the I_D_/I_G_ ratio, 0.55 in TDA, was very close to the BU. Therefore, apart from the size difference, the top-down and bottom-up cores were very similar.

To conclude the structural characterization, the nanodots were analyzed by ATR spectroscopy, which made it possible to determine the functional surface groups present on the different CDs. As evidenced by Figure 1f, the sample that presented the most complex surface structure was the BU, based on the high number of different signals which can be discerned in the ATR spectrum. In particular, BU displayed broad bands at about 3500 and 3200 cm^−1^ that can be attributed to OH and NH stretching vibrations, respectively, a small band at 2990 cm^−1^ due to CH stretching, a very strong C=O stretching signal located at 1718 cm^−1^, a C–N stretching vibration around 1400 cm^−1^, a small signal around 1197 cm^−1^, which can be attributed to C–O–C stretching, and several minor peaks of uncertain attribution. The C=O stretching signal most likely contains contributions from both amide carbonyl (CONH_2_), already present in urea, and carboxylic acid (COOH), left as a residue of citric acid.

The ATR spectra of the top-down CDs appeared much simpler than the BU, even after functionalization. The most prominent signals were found in the 1100–1200 cm^−1^ region, which can be attributed to C–O stretching vibrations. In particular, all the samples showed a prominent peak at 1120 ± 10 cm^−1^ due to C–OH surface groups vibrations. Only in the TDA did we detect an additional peak at 1193 cm^−1^, which can be attributed to C–O–C stretching vibrations. Thus, the functionalization with acetone led to the formation of plentiful C–O–C bonds on the surface. Besides the C–O vibrations, all three TD nanoparticles also showed the broad band at about 3450 cm^−1^, mostly due to OH stretching, and only the TDN showed significantly increased absorption in the region of N–H vibrations around 3200 cm^−1^, confirming its successful functionalization with amine (likely NH_2_) surface groups. Interestingly, no C=O stretching vibrations were visible in the infrared spectra of the TD samples.

Summarizing these results, both the bottom-up and top-down protocols lead to the successful production of CDs, defined as nanoparticles with a few nanometers diameter, a clear defective graphitic core, and a functionalized surface rich of oxygen. In this sense, the two types of CDs are very similar to each other. However, some key differences were highlighted as well in our study: the bottom-up CDs are substantially smaller and display a complex and highly polyfunctional surface dominated by C=O moieties; in contrast, the TD samples are larger in size and display a much simpler surface structure. The main surface groups introduced by functionalization are C–O–C and NH_2_ for TDA and TDN, respectively.

### 3.2. Optical Characterization

**Figure 2:**
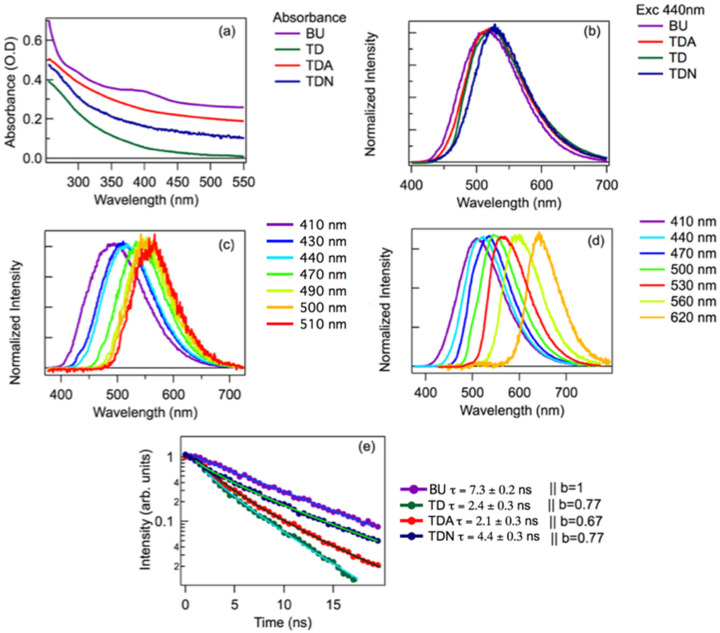
(**a**) Absorption spectra for the different CD samples. The spectra were vertically shifted for the sake of clarity. (**b**) Emission spectra normalized to the maximum of the different samples excited at the same wavelength of 440 nm. (**c**) BU emission tunability normalized to the maximum as observed exciting the sample in the range 410–510 nm. (**d**) TDA emission tunability normalized to the maximum exciting the sample in the range 410–620 nm. (**e**) Excited-state depopulation kinetic traces, as collected by time-resolved fluorescence measurements on the different CDs, excited at a wavelength of 440 nm.

Figure 2 summarizes the optical properties of the various types of CDs. The optical absorption (OA) spectra of all the TD samples, shown in Figure 2a, appeared broad and structureless across the entire wavelength range, suggesting a quasi-continuum of absorption transitions, almost independent of surface functionalization. The absorption spectrum of BU looked very different, with a well-defined absorption band peaking around 400 nm, suggesting a fundamentally different nature of the chromophores responsible for the absorption.

All the types of carbon dots emitted appreciable fluorescence under excitation across the visible range. In addition, the quantum yield (QY) for the four systems examined was calculated, exciting the samples at a wavelength of 440 nm, obtaining 46.4% for BU, 6.1% for TD, 9.2% for TDA, and 8.5% for TDN. Thus, as is generally known [1,6], the QY of BU-CDs is much higher than TD-CDs, and the QY of the latter can be enhanced by passivation. At a first glance (Figure 2b), in all samples the fluorescence appears as a broad band with maximum efficiency in the green when excited in the blue. Remarkably, very little differences appear among all samples when excited at the same wavelength (Figure 2b): in particular, the emission peaks at 515, 523, 524, and 526 nm for BU, TDA, TD, and TDN, respectively.

Besides, the fluorescence was highly excitation-dependent in all samples. In BU (Figure 2c) the peak continuously shifted from 490 to 530 nm when excitation went from 410 nm to 510 nm, with negligible changes of shape. Fluorescence from the TDA sample, shown in Figure 2d, has a similar trend, although the range of tunability was larger, and significant emission was still collected when exciting in the red tail of the very broad absorption spectrum (compare with Figure 2a). Emission lifetime analysis (Figure 2e) revealed an important difference between BU and TD samples. BU displayed a single-exponential kinetics with a lifetime of (7.3 ± 0.2) ns. In the TD samples, the kinetics looked markedly non-exponential and faster than the BU, confirming the substantially different emission mechanism for top-down and bottom-up carbon dots. The different functionalization led to appreciable changes between the TD samples. From a fit with a stretched exponential function I(t) = A exp [−(t/τ)^b^], we estimated lifetimes and stretching parameters finding τ = (2.1 ± 0.3) ns, b = 0.67 for TDA, τ = (2.4 ± 0.3) ns, b = 0.77 for TD, and τ = (4.4 ± 0.3) ns, b = 0.77 for TDN, respectively. The fluorescence tunability observed in Figure 2c,d is one of the most characteristic optical fingerprints of CDs, usually attributed to their surface disorder and size distribution [2]. This property was clearly observed in both BU and TD samples independently of the synthesis route, confirming a fundamental similarity between the different types of CDs. Indeed, Figure 2b–d is particularly representative in this respect, because the panels demonstrate that steady-state fluorescence alone would not allow us to clearly discriminate the BU from the TD samples nor to discriminate differently-passivated TD samples from each other. Because the size of TD and BU cores are different, the data also confirm that size is hardly relevant in determining the optical response of carbon dots, ruling out effects of quantum confinement. However, the time-resolved data and absorption spectra display some crucial differences that can be seen as fingerprints of two different fluorescence mechanisms for BU and TD samples, respectively.

From a phenomenological point of view, the optical properties we observed in BU can be described as “molecular-like”, in that the sample displayed well-defined optical absorption bands, an approximate excitation-emission mirror symmetry, and single exponential decays. Nevertheless, its emission displayed a significant tunability, a clear sign of a large structural disorder typically absent in molecular systems. Most likely, this disorder stems from dot-to-dot variations in surface structure, such as the presence of surface groups with slightly different local environments. Interestingly, BU dots did not display an enhanced degree of tunability when compared to TD, TDA, and TDN, despite the much more complex surface structure of the former. This finding is consistent with a recent suggestion [54] that the optical transition of bottom-up dots remains relatively localized on a small portion of the surface and specifically involves the very abundant COOH groups behaving as surface electron traps. In particular, in a recent work [54] we used steady-state and time-resolved optical methods, combined with density-functional theory, to demonstrate that the lowest-energy absorption transition of N-doped bottom-up carbon dots is due to a charge transfer transition from molecular orbitals occupied by nonbonding electrons of core N atoms to the empty acceptor molecular orbitals associated with surface COOH groups. As the transition is very localized, it can be described as having a “molecular” nature.

Yet, the idea of relating the emission mechanisms to a single surface group is most likely an oversimplification for a system of this complexity, as further confirmed by fluorescence quenching experiments shown in the following discussion.

In contrast, the optical response of TD appeared to be controlled by a quasi-continuum of absorption transitions. The highly non-exponential decays, almost independent of surface functionalization, are inherently non-molecular and more similar to what is typically observed in fluorescent nanoparticles [55]. This behavior points to “collective” electronic transitions that are rather delocalized on a wide portion of the dot. While their energy position was weakly influenced by local changes in the surface moieties (Figure 2b), it remained very sensitive to dot-to-dot long-range structural variations, which explains the high degree of fluorescence tunability of TD dots (Figure 2d). On the other hand, surface passivation has an appreciable influence on non-radiative decay patterns, showing up as changes in the QY and in the depopulation kinetics (Figure 2e).

### 3.3. Interaction with Mercury Ions

The samples can be further discriminated, and their properties analyzed on the basis of their response to mercury ions in solution. These sensing tests were performed on all four samples by studying the changes of the fluorescence observed in the presence of increasing amounts of mercury (Hg^2+^). Because the electrochemical potential for the reduction of mercury is very low (0.85 V vs. standard hydrogen electrode), one expects mercury to easily quench the fluorescence by an electron transfer from the emissive chromophores of CDs to Hg^2+^ ions, provided that the ion finds suitable interaction sites at CD surfaces. Thus, because the redox potential should not be the limiting factor of fluorescence quenching, Hg^2+^-induced quenching provides a convenient probe of the different interaction sites on the various types of CDs and their interplay with the emissive chromophores.

**Figure 3:**
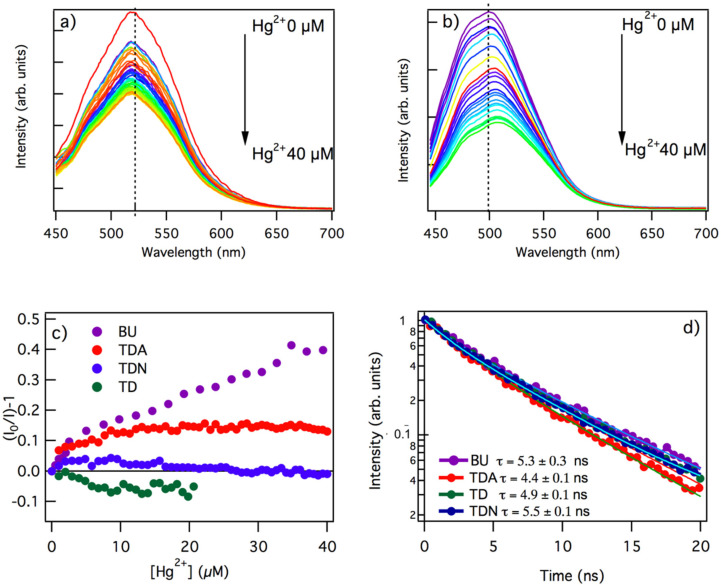
Emission spectra under excitation at 440 nm of the (**a**) TDA and (**b**) BU samples in aqueous solution in the presence of increasing concentrations of Hg^2+^ ranging from 0 to 40 µM. (**c**) Stern–Volmer quenching plots of the integrated emission under excitation at 440 nm of BU, TDA, TD, and TDN samples at different concentrations of Hg^2+^; I_0_ and I represent the emission intensity at zero and non-zero concentrations of Hg^2+^, respectively. (**d**) Excited-state decay kinetics of the emission under excitation wavelength of 440 nm of BU, TDA, TD, and TDN samples in the presence of the same concentration of 40 µM mercury.

As shown in Figure 3, we found that samples BU and TDA both displayed a significant response to Hg^2+^, with a decrease of the emission intensity clearly measurable upon the addition of even a few µM Hg^2+^ ions. In contrast, we found that neither the non-functionalized TD sample, nor the amine-functionalized TDN sample were quenched by mercury ions within the experimental errors. Exciting the TDA sample at 440 nm and increasing the concentration of mercury (Figure 3a), the sample presented a quenching already at the lowest tested concentration (1 µM). Successive additions led to a further reduction in the emission intensity, which leveled to about 60% of the initial value after the addition of 40 µM ions. Here, quenching occurred with no significant changes of the emission band-shape.

The behavior of BU, shown in Figure 3b, was very different. On the one hand, the extent of quenching was markedly higher, and the intensity of the emission decreased to about 40% after the addition of 40 µM mercury ions. Besides, in addition to the quenching, the fluorescence underwent a red shift, whereby the spectral maximum of the band moved from 522 nm to 534 nm when increasing the Hg^2+^ concentration. This change of shape is clearly most advantageous for sensing applications because it provides the emission peak position as an additional observable to monitor Hg^2+^, along with fluorescence intensity.

Stern–Volmer plots of the fluorescence data (Figure 3c) confirmed that the bottom-up and top-down samples responded differently to mercury ions. In TDA, after a very marked initial quenching already at low concentrations, the Stern–Volmer curve quickly reached a plateau. This suggests the complete saturation of a specific functional group on which mercury selectively, and very effectively, binds at the CD surface. Considering that no quenching was observed in the non-functionalized TD and in the TDN samples (see Figure 3c), and based on the surface properties inferred from ATR (Figure 1), it is very likely that C–O–C moieties on the surface are the preferred binding site for Hg^2+^ ions, because C–O–C were only detected in TDA and absent in TD and TDN (see Figure 1f). While we expected a strong interaction between the negative charge on oxygen and nitrogen lone pairs and the positively charged Hg^2+^ ions, our data suggest that the affinity of mercury ions to C–O–C moieties is much higher than both C–OH and C–NH_2_ groups found on TD and TDN, respectively.

The Stern–Volmer plot of the BU sample was very different and displayed two distinct trends with different slopes, one at low concentrations (from 0.5 to 5 µM) and one at higher concentrations (>5 µM), the latter accounting for the majority of quenching. This suggests at least two different functional groups on the surface of the dot that may coordinate covalently with mercury, whereby interaction with the second group (at higher concentration) becomes appreciable only after saturation of the first. Generally speaking, the much stronger quenching exhibited by BU is consistent with the highly functionalized surface, which certainly hosts abundant binding sites for Hg^2+^. Besides, a certain heterogeneity of binding sites at the surface of BU is to be expected considering the highly polyfunctional nature of their surfaces (Figure 2) and may also explain the progressive red shift of the fluorescence (Figure 3b), if one assumes that the addition of Hg^2+^ selectively “turns off” a subset of the possible emission chromophores and progressively emphasizes the radiative channels responsible for a red-shifted emission.

The strongest contribution to BU fluorescence quenching ([Hg^2+^] > 5 µM) is most likely associated with the interaction of mercury ions and C=O groups, very abundant on the surface (see Figure 1f) and closely involved in the emission mechanism of these dots [5,7,8,24,28]. The initial smaller variation seen in the Stern–Volmer plot, observed at low concentrations, must be attributed to another surface moiety, being much less abundant than C=O, but strongly interacting with mercury ions. Besides C=O groups, BU samples also host a certain concentration of superficial C–O–C groups, as shown in Figure 1f. Therefore, by analogy with TDA samples, we propose that the earliest trend observed in the Stern–Volmer plot of BU samples is associated with the interaction of Hg^2+^ with C–O–C sites.

Finally, the lifetime of the CDs taken into consideration was measured (Figure 3d) after the addition of a concentration 40 µM of mercury ions. In general, lifetime analysis is a useful tool to discriminate static from dynamic quenching [49]. Indeed, considering the very low concentration of Hg^2+^ ions at play, one suspects that the quenching mechanism here cannot be diffusional, but must be essentially static; that is, quenching occurs through the formation of a stable coordination compound of mercury with surface functional groups on CDs.

From our measurements, we found that the BU sample had a lifetime of 5.4 ns in presence of Hg^2+^. Although this was lower than the lifetime in the absence of mercury (7.3 ns), the reduction did not match the decrease in intensity (40% at the same concentration of Hg^2+^, as from Figure 3b,c), thus indicating that at least a part of the quenching occurs through a static mechanism [38,43,48]. Considering the top-down samples, we found a lifetime of 4.7 ns for the TD, 4.4 ns for the TDA, and 4.8 ns for the TDN, which were longer than the values without mercury (2.4, 2.1, and 4.4 ns, respectively). Additionally, this result is consistent with a static quenching, assuming that the increase in lifetime is likely from the extinction of non-radiative channels due to the interaction with the solvent, which is obtained through electrostatic screening due to the presence of mercury ions. In fact, a similar effect was found before for bottom-up CDs interacted with Zn^2+^ ions [39].

From a practical point of view, these data demonstrate the need of oxygen-rich groups such as C=O and C–O–C, suggested here as the active binding sites on the surface of CDs, in order to get a significant response to quencher ions. Even after deliberate functionalization, which is crucial to activate a quenching response in TD samples, the abundance of binding sites on the latter remains relatively low as compared to BU, which are inherently very rich of C=O groups right after synthesis. For these reasons, BU samples behave much better as sensors, with the additional benefit of a progressive change of shape of the emission band with increasing Hg^2+^ ions, which can be seen as a side effect of their highly complex surface. On the other hand, the lack of sensitivity of TD and TDN surfaces might make them more appropriate for different applications, whenever ease of quenching is an undesired feature.

## 4. Conclusions

A side-to-side comparison between typical bottom-up and top-down CDs confirms they share a range of essentially similar morphological and optical properties, such as a well-defined carbonaceous core with a defective graphitic structure, which is directly confirmed by the D and G Raman lines at 1356 and 1590 cm^−1^, respectively, a tunable fluorescence with very similar band-shape in the two types of CDs, and the fluorescence quenching by micromolar concentration of mercury ions, which makes them useful for sensing applications. These findings endorse a description of bottom-up and top-down CDs as two sub-types of the same family of nanomaterials. On the other hand, deeper analysis highlights several key differences between TD and BU carbon dots, providing practical protocols to discriminate them from each other. TD nanoparticles display a quasi-continuum of optical absorption transitions, a complete lack of absorption-emission mirror symmetry, and non-exponential emission decays with lifetimes ranging from 2.1 to 4.4 ns, suggestive of highly delocalized electron transitions. On the other hand, their surface structure is relatively simple, and their interaction with electron acceptor ions is characterized by single-step quenching on a single surface site if an appropriate interaction site is provided by suitable functionalization. The emission of these dots is quenched to about 65% of the initial value in the presence of 40 µM Hg^2+^ ions. On the other hand, BU samples emit through molecular-like electronic transitions that are relatively localized on the surface. The main optical fingerprints of these transitions are an absorption band at 400 nm, which excites a tunable emission decaying with a lifetime of 7.3 ns. The surface structures of BU dots are much more complex and highly polyfunctional than TD, leading to a stronger interaction with quenchers, with the emission being quenched to about 40% by 40 µM Hg^2+^ ions. Another interesting aspect of our findings is that the fluorescence quenching of BU samples by mercury is accompanied by a progressive redshift of the fluorescence, which is particularly suitable for practical applications. Such a result may be useful for the further technological and practical developments in the field of CD-based sensing. In this respect, we carried out some additional quenching tests (not shown) of both TD and BU carbon dots with several metal ions, and we found that, despite the fact that several common ions such as Cu^2+^ and Fe^3+^ are capable of inducing emission quenching of the emission of both types of CDs, the fluorescence redshift that accompanies quenching is only observed for BU dots interacting with mercury ions, a property which may open a very convenient route for the selective detection of these ions by using BU carbon dots as sensors.

## Data Availability

The data presented in this study are available on request from the corresponding author.
